# A Novel Vaccine against Crimean-Congo Haemorrhagic Fever Protects 100% of Animals against Lethal Challenge in a Mouse Model

**DOI:** 10.1371/journal.pone.0091516

**Published:** 2014-03-12

**Authors:** Karen R. Buttigieg, Stuart D. Dowall, Stephen Findlay-Wilson, Aleksandra Miloszewska, Emma Rayner, Roger Hewson, Miles W. Carroll

**Affiliations:** Microbiology Services Research, Public Health England, Porton Down, Wiltshire, United Kingdom; University of Liverpool, United Kingdom

## Abstract

Crimean-Congo Haemorrhagic Fever (CCHF) is a severe tick-borne disease, endemic in many countries in Africa, the Middle East, Eastern Europe and Asia. Between 15–70% of reported cases are fatal. There is no approved vaccine available, and preclinical protection *in vivo* by an experimental vaccine has not been demonstrated previously. In the present study, the attenuated poxvirus vector, Modified Vaccinia virus Ankara, was used to develop a recombinant candidate vaccine expressing the CCHF virus glycoproteins. Cellular and humoral immunogenicity was confirmed in two mouse strains, including type I interferon receptor knockout mice, which are susceptible to CCHF disease. This vaccine protected all recipient animals from lethal disease in a challenge model adapted to represent infection via a tick bite. Histopathology and viral load analysis of protected animals confirmed that they had been exposed to challenge virus, even though they did not exhibit clinical signs. This is the first demonstration of efficacy of a CCHF vaccine.

## Introduction

A new tick-borne disease, Crimean Haemorrhagic Fever, was first described in the Crimea in 1945 [1]. In 1969 it was recognised that the virus causing the disease was identical to a virus isolated in the Congo in 1956 [2], and the disease was subsequently renamed Crimean-Congo Haemorrhagic Fever (CCHF). The causative agent, CCHF virus (CCHFv), is a member of the *Nairovirus* genus in the *Bunyaviridae* family.

People may be infected with CCHFv by the bite of infected *Ixodidae* ticks, contamination with tick body contents, or direct contact with the blood, tissues or body fluids of infected human beings or animals. The most efficient and common tick vectors appear to be members of the *Hyalomma* genus, which commonly infest livestock and other animals. CCHF causes a severe human disease, with a fatality rate of 15–70% [3]. Due to the haemorrhagic nature of the disease, it can also cause nosocomial infections of health-care workers [4]. Although clinical predictors of mortality have been developed [5], correlates of protection are not fully understood. Detectable antibody responses are usually required for survival, but are not predictive of recovery [6]. Therefore it is likely that both antibody and CD8-positive T lymphocytes are required for protection.

CCHF is endemic in many countries in Asia, the Middle East, Eastern Europe and southern Africa, and it is an increasing problem for Western Europe, including popular tourism destinations such as Greece and Turkey [7]. The incidence and geographic range of CCHF is increasing, and non-endemic countries are also at risk from imported cases; in October 2012, a fatal case of CCHF occurred in a traveller returning to the UK from Afghanistan [8]. Additionally, concerns over the potential use of CCHFv as a bioterrorism agent have led to the US National Institute of Allergy and Infectious Diseases to list the virus as a Category C priority pathogen. Due to the severity of disease, ease of transmission, and the fact that no antiviral compounds or vaccines have been proven to be effective against it, CCHFv is classified as a hazard group 4 pathogen by the UK Advisory Committee on Dangerous Pathogens.

Progress with CCHFv research has been severely hampered by the lack of a suitable animal model. Newborn mice succumb to infection [9], but due to their immature immune systems, they cannot be used for assessing vaccine efficacy. Recently, however, adult small animal models have been developed, using mice deficient in the type I interferon (IFN) signalling pathway, either in the type I IFN receptor (IFNα/βR) [10], [11] or in STAT-1 [12]. CCHFv infection in IFNα/βR-knockout mice replicates human disease *via* a variety of inoculation routes, including subcutaneous injection which is intended to mimic human infection by tick bite [11].

A vaccine based on CCHFv, amplified in suckling mouse brain and inactivated by chloroform treatment, has been used in Eastern Europe, but is unlicensed by the European Medicines Agency or US Food and Drug Administration. A recent study found that it elicited both a cellular and humoral response to CCHFv, but neutralising antibody titres were low, even in people who had received 4 doses [13]. Controlled studies on protective efficacy have not been reported with this vaccine and, due to its crude preparation, it is unlikely to gain widespread international regulatory approval. Development of a vaccine that meets current international standards is needed. The genome of CCHFv is distributed over three RNA segments: small (S), medium (M), and large (L), which encode the viral nucleoprotein, glycoprotein and RNA polymerase, respectively. The M segment is approximately 5.4 kbp in length and encodes a polyprotein precursor that undergoes proteolytic cleavage events [14], [15]. It contains a single open reading frame of 1685 residues (in strain IbAr10200), which is processed into mature glycoproteins G_N_ and G_C_, and three other domains of as-yet unknown function: a variable mucin-like domain, a GP38 domain, and an NSm domain. The G_N_ and G_C_ glycoproteins of CCHFv bind target cells and influence infectivity [16] and cell tropism, and are the targets for neutralising antibodies [17]. Virus entry is thought to be primarily mediated by G_C_, which binds the proposed virus receptor human cell surface nucleolin [18].

Recent vaccine approaches for CCHF include a DNA-based vaccine expressing the CCHFv M segment, which induced neutralising antibodies in approximately half of vaccinated mice, but the lack of a challenge model at the time meant efficacy could not be demonstrated [19]. Another vaccine candidate using transgenic tobacco leaves expressing G_N_ and G_C_ was fed to mice and induced IgG and IgA. However, antibodies were not tested for neutralisation titres and this oral vaccine was not tested for protection [20]. Neither of these studies investigated cellular immune responses. There is, therefore, at present no safe and effective, commercially-available vaccine against CCHFv.

Poxviral vectored vaccines, such as those based on Modified Vaccinia virus Ankara (MVA), have the capacity to accommodate large gene inserts [21], such as the CCHFv M segment. Despite its growth deficiency in most mammalian cells [22], [23], MVA is able to promote high-level gene expression of recombinant genes both *in vitro* and *in vivo*, with authentic post-translational modifications in the host cell. Both humoral and cellular immunity [24], [25] can be induced by MVA, without the requirement for an adjuvant.

We aimed to develop a candidate vaccine based on recombinant MVA expressing the CCHFv glycoproteins, to assess the induction of cellular and humoral immunity, and to evaluate efficacy in a challenge model that represents human disease. We adapted a previously described animal model to mimic infection by tick bite, by establishing intradermal delivery of CCHFv to IFNα/βR deficient mice. This is the first demonstration of a CCHF vaccine candidate conferring protection in a preclinical model.

## Materials and Methods

### Animals

Female A129 (IFNα/βR^-/-^) and 129Sv/Ev mice, aged 5-8 weeks, were obtained from B&K Universal (UK). Immunocompromised animals were housed in an aseptic environment to protect them from opportunistic infections. All efforts were made to minimise animal suffering and endpoints were limited to a moderate severity rating. These studies were approved by the ethical review process of Public Health England, Porton Down, UK and the Home Office, UK, *via* project licence numbers 30/2476 and 30/2697. Work was performed in accordance with the Animals (Scientific Procedures) Act 1986 and the Home Office (UK) Code of Practice for the Housing and Care of Animals Used in Scientific Procedures (1989).

### Cells

BHK-21 cells (CCL-10) were obtained from ATCC (USA) and cultured in modified essential eagle medium (Sigma-Aldrich, USA) supplemented with 10% foetal bovine serum (FBS), 2 mM L-Glutamine, 100 U penicillin & 0.1 mg/ml streptomycin (Sigma-Aldrich). CEF cells were obtained from the Institute for Animal Health (Compton, UK) and cultured in Dulbecco's modified eagle medium (Sigma-Aldrich), supplemented as above. SW13 (accession number 87031801) and Vero E6 (accession number 85020206) cells were obtained from the European Collection of Cell Cultures, UK. Cultures were maintained in Leibovitz's L-15 medium containing Glutamax (Life Technologies, UK) supplemented with 10% FBS.

### Viruses

MVA (strain MVA 1974/NIH clone 1) was kindly supplied by Prof B. Moss (NIH, USA). Virus titre was determined by plaque assay [26] in BHK-21 cells. CCHFv (strain IbAr10200) was amplified in suckling mouse brain and homogenised with a disposable pestle and mortar. Titre was determined by TCID_50_ in Vero E6 cells.

### Construction of plasmids

A cassette containing Gateway system attR1 and attR2 recombination sequences (Life Technologies, USA; [27]) was generated. The upstream side was adjacent to a Kozak sequence and start codon followed by the human tissue plasminogen activator (tPA) leader sequence (36 amino acid residues). The downstream side was adjacent to an 8 residue linker sequence followed by the 14 residue V5 epitope [28] and a stop codon. This cassette was inserted between the *XmaI* and *SalI* restriction sites of plasmid pLW-44 [29] (kindly provided by Prof B. Moss, NIH) to produce plasmid pDEST44-TPA-V5.

The M segment nucleic acid sequence of CCHF strain IbAr10200 (Accession number U39455) was modified by removal of untranslated regions, initiation and termination codons. To enable Gateway recombination, attB1 and attB2 sequences were added to the beginning and end, respectively, of the remaining open reading frame (i.e. nucleotides 34 to 5082). Two poxvirus transcription stop signals (TTTTTNT) at positions 3894 and 4767 were altered without introducing coding changes. The resulting sequence was synthesised and then recombined into pDONR (Life Technologies) by Entelechon (Germany), to generate plasmid pENTR-GP.

Plasmids pDEST44-TPA-V5 and pENTR-GP were recombined using Gateway technology to generate plasmid pTP-GP.

### Generation of recombinant MVA expressing CCHFv glycoprotein

BHK-21 cells were infected with MVA 1974 at a multiplicity of infection of 0.05. Infected cells were transfected with pTP-GP using Lipofectamine (Life Technologies) as directed by the manufacturer. The resulting recombinant MVA-GP was serially plaque-purified 4 times in BHK-21 cells, based on green fluorescent protein (GFP) expression. MVA-GP was amplified on BHK-21 and CEF cells, purified by sucrose cushion centrifugation [30] and titrated by plaque assay [26] on BHK-21 cells, prior to use in *in vivo* studies. Plaques were visualised using GFP fluorescence or by immunostaining [26] with rabbit anti-Vaccinia antibody (AbD Serotec, UK) and Vectastain Universal ABC-AP kit (Vector Laboratories, USA). Genomic DNA from infected cells was extracted using the Wizard SV genomic DNA purification system (Promega, USA) and used as a template in PCR with AccuPrime Taq DNA polymerase High Fidelity (Life Technologies) for genotype analysis.

### MVA-GP vaccination

Groups of 5–12 mice aged 5–8 weeks were injected intramuscularly into the caudal thigh with 10^7^ plaque-forming units (pfu) per animal of MVA-GP diluted in endotoxin-free PBS. Control animals received 10^7^ pfu/animal of non-recombinant MVA 1974 or an equivalent volume of saline. A total volume of 100 μl was delivered to each animal across two sites, each with 50 μl. Animals received a booster vaccination 14 days later. Animals were euthanised and tissues were collected 21 or 28 days after the primary vaccination.

### IFN-γ ELISpot assay

Spleens from vaccinated animals were collected aseptically, homogenised, and red blood cells were lysed. Splenocytes were resuspended in RPMI medium (Sigma-Aldrich) supplemented with 5% FBS, 2 mM L-Glutamine, 100 U penicillin & 0.1 mg/ml streptomycin, 50 μM 2-mercaptoethanol and 25 mM HEPES solution (Sigma-Aldrich). Splenocytes were assessed for antigen recall response via IFN-γ ELISpot (Mabtech, Sweden), performed as per the manufacturer's instructions. Cells were seeded in PVDF microtitre plates at 2×10^5^ per well and re-stimulated with peptide pools (Mimotopes, Australia). Peptides spanning the tPA-GP-V5 fusion protein sequence were 20 residues long, with an overlap of 12 residues between peptides. They were applied to cells at a final concentration of 2.5 μg/ml per peptide, with 28–32 peptides per pool. Plates were developed after 18 hours at 37°C, 5% CO_2_ in a humidified incubator. Spots were counted visually on an automated ELISpot reader (Autoimmun Diagnostika, Germany). Background values from wells containing cells and medium but no peptides, were subtracted and pools summed across the target protein. Results were expressed as spot forming units (SFU) per 10^6^ cells.

### Western blot

For use in Western blot analysis, SW13 cell monolayers were infected with CCHFv at a multiplicity of infection of 0.01, and incubated at 37 °C in Leibovitz's L-15 medium containing 2% FBS. The medium was removed 48 hours post-infection and the cells were treated with Laemmli buffer supplemented to contain 10% sodium dodecyl sulphate (Sigma-Aldrich). The resultant mixture was collected into vials and heat treated at >90 °C for at least 10 minutes. Uninfected SW13 monolayers were treated similarly, as a negative control.

Sucrose cushion-purified MVA, or lysates from CCHFv-infected, or uninfected, SW13 cells were subjected to SDS-PAGE [31] on a 4–12% Bis-Tris gel (Life Technologies) and proteins were transferred to a PVDF membrane. After blocking in 5% milk protein (Oxoid, UK), membranes were incubated with primary antibody for 1–2 hours, washed 6 times with PBS containing 0.05% Tween-20 (Sigma-Aldrich), or 0.05% NP-40 (Sigma-Aldrich), incubated for 1 hour with HRP-conjugated secondary antibody and washed as before. Bound antibody was detected with ECL-Prime WB Detection reagent (GE Life Sciences, USA) according to the manufacturer's instructions and visualized on a ChemiDoc system (BioRad, USA). Molecular weights were calculated by comparison with markers of known molecular weight using QuantityOne software.

Primary antibodies used were mouse anti-V5 (AbD Serotec) diluted 1/5000, polyclonal rabbit serum (kindly provided by A. Mirazimi, Sweden) raised against glycoprotein peptides and diluted 1/2500, or serum from vaccinated mice diluted 1/1000. Secondary antibodies were anti-mouse IgG (Sigma-Aldrich) diluted 1/8000, anti-mouse IgG/A/M (AbD Serotec) diluted 1/4000, or anti-rabbit IgG (Sigma-Aldrich) at 1/5000.

Where the primary antibody was serum from vaccinated mice, all dilutions were made in PBS containing 0.05% NP40 and 5% milk protein. For all other Western blots, dilutions were made in PBS containing 0.05% Tween-20 and 5% milk protein.

### Enzyme-linked immunosorbent assay (ELISA)

Recombinant G_N_ ectodomain, expressed in a mammalian system, was kindly provided by Dr Thomas Bowden (University of Oxford, UK). This was diluted in 0.05 M carbonate-bicarbonate buffer pH 9.6 (Sigma-Aldrich), and used to coat Maxisorp 96-well plates (Nunc, Denmark) at 200 ng/well in a volume of 100 μl/well. Plates were covered with plate sealers and incubated overnight at 4°C. They were washed twice with 300 μl/well of PBS 0.05% Tween-20 using an AquaMax (Molecular Devices, USA) plate washer. Plates were blocked with 100 μl/well of 5% skimmed milk powder in PBS 0.05% Tween-20 for 1 hour at 37°C with shaking, and then washed three times. Samples, diluted in 5% skimmed milk powder in PBS 0.05% Tween-20, were added to plates at 100 μl/well in triplicate and incubated for 1 hour at 37°C with shaking. Normal mouse serum (Sigma-Aldrich) was used as a negative control sample. Plates were washed three times before the addition of 100 μl/well of polyclonal HRP-conjugated goat anti-mouse IgG/A/M (AbD Serotec), diluted 1/4000 in 5% skimmed milk powder in PBS 0.05% Tween-20. Alternatively, HRP-conjugated goat anti-mouse IgM (Sigma-Aldrich), diluted 1/5000, was used. After 1 hour incubation at 37°C with shaking, plates were washed three times.

ABTS ELISA HRP substrate (KPL, USA) was prepared according to manufacturer's instructions and 100 μl/well was added. After incubation at room temperature for 25 minutes with shaking, the reaction was stopped with 100 μl/well of ABTS peroxidase stop solution (KPL), prepared according to manufacturer's instructions. Optical density was measured at 405 nm with a Spectramax M3 Multimode Spectrophotometer (Molecular Devices). A 4 parameter-logistic curve fit was plotted for each sample in SoftMax Pro version 5.4 (Molecular Devices). The endpoint titre was the interpolated dilution at an absorbance of 0.250. The ratio of expected to observed endpoint titres of an internal reference sample, was used as a correction factor to compare samples across multiple plates.

### CCHFv challenge of A129 mice

A129 mice received 200 TCID_50_ CCHFv, unless otherwise stated, in a volume of 100 μl, intradermally in the upper medial area of the back.

Subsequently, animals were weighed and monitored for body temperature daily. In addition, they were observed for clinical signs twice daily. Signs of illness were converted to a numerical score as follows: normal  =  0, arching or ruffling  =  1, arching and ruffling  =  2, lethargy  =  3, immobility  =  4. Animals showing moderate signs (e.g. loss of 10% body weight, lethargy or immobility) were euthanised. At 4 days post-challenge, randomly selected surviving animals were killed humanely and samples of blood, spleen and liver were collected for viral load studies, and spleen and liver for histopathological examination. At 14 days post-challenge all surviving animals were killed humanely and samples were collected as at 4 days post-challenge. In addition, approximately 0.5 cm^3^ sections of liver and spleen were harvested aseptically and stored at −80 °C for subsequent viral amplification. Any animals that were euthanised at any other time point, had spleen and liver samples collected for histopathological examination only.

### Pathological studies

Samples for histopathological examination were placed in 10% neutral buffered formalin for 7 days, and processed routinely to paraffin wax. Sections were cut at 5–6 μm, stained with haematoxylin and eosin (HE) and examined microscopically.

For immunohistochemistry, formalin-fixed, paraffin-embedded sections of spleen and liver, cut at 4 μm, were mounted on positively charged X-tra Adhesive slides (Leica Biosystems, UK), deparaffinised and rehydrated. Immunohistochemical staining was achieved using a BOND-MAX Immunostainer (Leica Microsystems, UK) and a Novacastra Bond Intense R (Leica Biosystems) detection kit. A heat-induced epitope retrieval cycle with buffer ER1 R (Leica Biosystems) was performed for 10 minutes. Slides were incubated with rabbit serum (4%) (Abcam, Cambridge, UK) for 20 minutes followed by an avidin/biotin blocking stage (15 minutes each) (Abcam). Polyclonal antibody raised in sheep immunised against recombinant CCHFv nucleoprotein (kindly provided by Dr John Barr, University of Leeds, UK) was incubated with the tissue for 30 minutes, followed by a biotinylated rabbit anti-sheep polyclonal antibody (Abcam) at a dilution of 1∶500, for 10 minutes. Haematoxylin was used as the counterstain. Positive and negative control slides were included. Immunolabelled slides were evaluated using light microscopy.

### Quantification of viral load by RT-PCR

Whole blood (100 μl) was collected into RNA Protect Animal Blood tubes (Qiagen, Netherlands) and stored at −80°C. Tubes were thawed, inverted and left for a further 2 hours at room temperature to ensure efficient cell lysis. Samples were treated with Red Blood Cell Lysis Solution (Miltenyi Biotec, Germany) before purification of total RNA using an RNeasy Mini kit (Qiagen).

For viral load analysis, spleen and liver samples were collected into RNALater (Qiagen) and stored at −80°C. Thawed tissue was transferred to RLT buffer (Qiagen), homogenised by passing through a 70 μm sieve and then treated using an RNeasy Mini kit (Qiagen) for extraction of total RNA.

CCHFv S segment was detected by RT-PCR on the ABi 7500 RT-PCR platform as described [32], with adjusted cycling conditions: 50°C for 20 minutes, 96°C for 5 minutes, followed by 45 cycles of 95°C for 15 seconds and 60°C for 30 seconds (with quantification analysis of fluorescence performed at the end of each 60°C step), and final cooling of 40°C for 30 seconds.

Each sample was also analysed for levels of hypoxanthine guanine phosphoribosyl transferase (HPRT) housekeeping gene. A one-step RT-PCR with singleplex detection was performed targeting an 89 bp product in the mouse HPRT gene (NCBI Reference sequence NM_013556) using the QuantiFast probe assay (Qiagen) and the ABi 7500 RT-PCR platform. All reactions were run in triplicate.

To normalise the CCHFv expression data, C_T_ values for CCHFv and HPRT were each inverted by subtracting the C_T_ value from 45 (the total number of cycles), where C_T_ is the number of cycles required to reach the fluorescence threshold value. The mean value of CCHFv was then divided by the mean value of the HPRT reference gene for each sample.

### Viral amplification from tissue samples

Tissues were thawed and homogenised through a 500 μm mesh with 2 ml Leibovitz's L-15 medium containing Glutamax supplemented with 2% FBS. Supernatant was clarified by centrifugation at 1500×*g* for 7 minutes before 50 μl was added to a 25 cm^3^ flask of SW13 cells. A clinical isolate of CCHFv was used as a positive control. Any virus present was left to absorb for 30 minutes, before 5 ml of medium was added to each flask. After incubation for 2 days at 37°C, cells were observed microscopically for cytopathic effects.

### Statistical and in silico analysis

Due to the data being distributed non-parametrically, the Mann-Whitney U test was performed to determine statistical significance between groups. Data analysis was conducted using GraphPad Prism software (Version 5.01, GraphPad Software, USA).

Predicted molecular weights of proteins were calculated using EditSeq software (Version 10.0.1, DNASTAR, USA).

## Results

### Construction of plasmids

**Figure 1:**
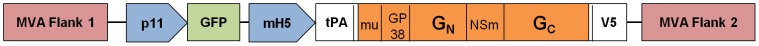
Schematic representation of the vaccine vector. Nucleotides 34 to 5082 of the CCHFv M segment (orange) were fused at the amino-terminus to the 36 amino acid residue tPA leader sequence, via 11 residues derived from the Gateway recombination process. The carboxy-terminus was fused to the 14 amino acid residue V5 epitope, via 11 residues of Gateway sequence, and 8 residues of a linker peptide. This product was under the control of the mH5 promoter, and between MVA flanks for insertion into the Deletion III site of the MVA genome. The insertion cassette also contained the enhanced GFP gene downstream of the p11 promoter, for identification of recombinant virus. The mucin-like domain is represented by ‘mu’.

Transfer plasmid pTP-GP was prepared as described in Materials & Methods. It encoded a tPA-GP-V5 fusion protein 14 nucleotides downstream of the mH5 poxvirus promoter [33] and flanked by MVA genomic sequences for insertion into the Deletion III region [34], [35] of MVA. pTP-GP also encoded the enhanced GFP gene downstream of the p11 poxvirus promoter for insertion into the same locus (Fig 1). The sequence of the inserted cassette is given in File S1.

### Generation of MVA-GP vaccine

Plasmid pTP-GP was used to generate a recombinant MVA virus, named MVA-GP, expressing the full length M segment open reading frame. The glycoprotein was fused to the signal sequence of tPA at the amino-terminus for increased immunogenicity and intracellular transport [36], [37]. The V5 epitope [28] was fused to the carboxy-terminus by a short linker sequence for immunodetection of *in vitro* protein expression. A total of 22 residues were also incorporated into the final recombinant gene product as a footprint of the attB sequences used during Gateway recombination. MVA-GP also expressed GFP. Enumeration of plaques by GFP visualisation compared to immunostaining with anti-Vaccinia antibody confirmed absence of GFP-negative virus.

PCR of the insert site produced a single, recombinant-specific product, and failed to amplify a parent-specific product (data not shown), indicating absence of parental MVA. Sequencing the insertion site confirmed that no spontaneous mutations in the transgene had been introduced.

**Figure 2:**
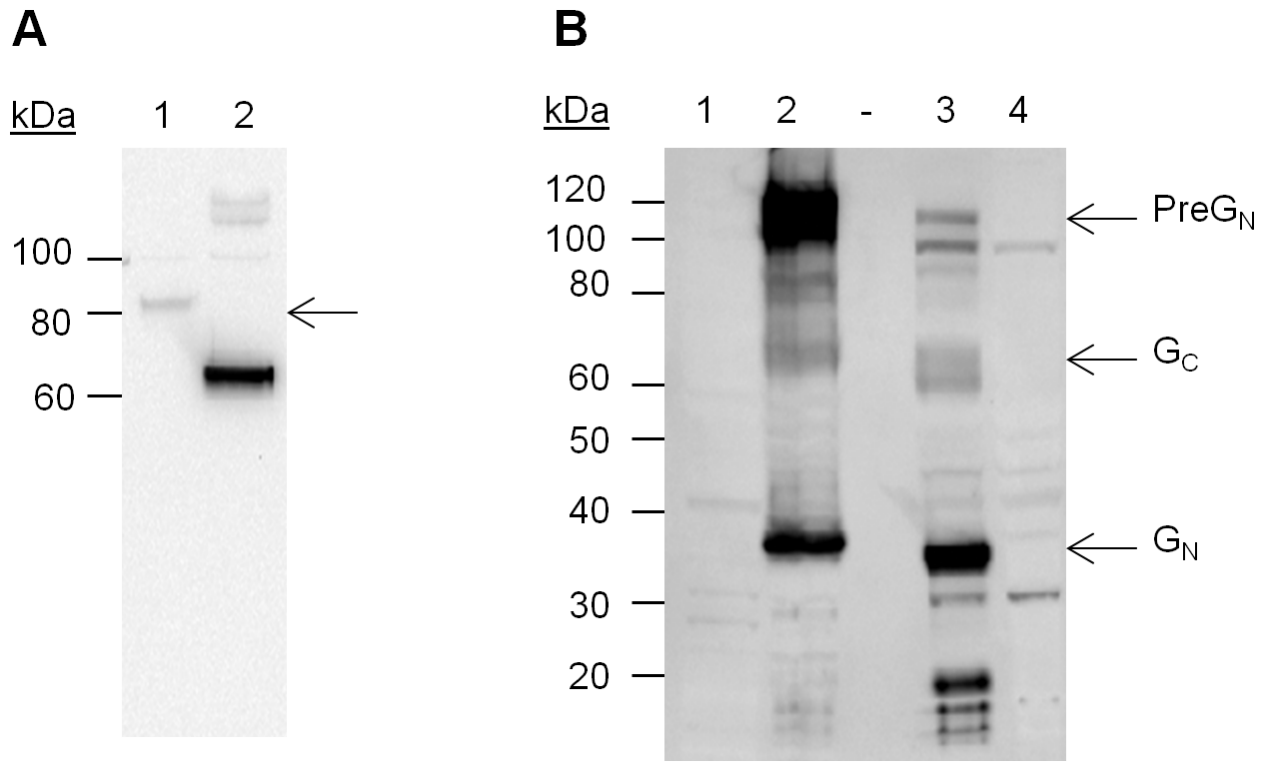
Western blot analysis of glycoproteins expressed by MVA-GP and CCHFv. (A) Western blotting of MVA-GP (lane 1) with anti-V5 antibody indicated a protein of approximately 75 kDa (arrow), confirming recombinant protein expression, and consistent with cleavage of the predicted 76.6 kDa G_C_-V5 fusion protein. A positive control (lane 2) expressed a V5 fusion protein of 62 kDa. (B) Western blot of MVA-1974 (lane 1), MVA-GP (lane 2), CCHFv-infected SW13 cells (lane 3) and uninfected SW13 cells (lane 4) with rabbit polyclonal antiserum. Major products expressed by MVA-GP corresponded with those expressed by CCHFv, indicating the recombinant protein underwent similar post-translational cleavages to the native protein. Arrows indicate processed glycoproteins at 109, 64 and 35 kDa. These are most likely to be PreG_N_, G_C_, and G_N_, respectively.

SDS-PAGE of MVA-GP and Western blotting with anti-V5 antibody (Fig 2A) indicated a single protein of approximately 75 kDa, confirming expression and consistent with cleavage of the predicted 76.6 kDa Gc-V5 fusion protein from the GP precursor at the RKPL sequence [14].

SDS-PAGE and Western blotting with anti-glycoprotein polyclonal serum were performed, in order to compare glycoprotein expression by MVA-GP with CCHFv (Fig 2B). Several products were expressed by CCHFv (lane 3). Some of these were also detected in uninfected SW13 cells (lane 4), suggesting a possible cross-reaction with cellular proteins. CCHFv-specific products were detected at approximately 109, 60, 34 and <20 kDa. Similarly, sucrose cushion-purified MVA-GP expressed major proteins at approximately 92–136, 64 and 35 kDa (lane 2). Sucrose cushion-purified MVA 1974 (a negative control) did not show any products of a similar size (lane 1), confirming all products in lane 2 as specific to the recombinant vaccine. Major products expressed by MVA-GP are most likely to be PreG_N_ (a G_N_ precursor), G_C_, and G_N_ with sizes of 109, 75 and 37 kDa, respectively [38].

A slight shift towards higher molecular weight proteins was observed in G_C_ expressed by MVA-GP, compared to that expressed by CCHFv. This may be explained by the V5 epitope tag, which adds 3.6 kDa. It is unclear why CCHFv-expressed G_C_ and G_N_ appear to be of a lower molecular weight than previously reported [38].

### Immunogenicity of MVA-GP

The ability of MVA-GP to induce a GP-specific IFN-γ cellular response was tested in 5-8 weeks old A129 mice, and in the wild-type parental strain, 129Sv/Ev. Mice (5 per group) were vaccinated with 10^7^ pfu/animal MVA-GP or MVA 1974, or an equivalent volume of saline, at day 0 and day 14. At day 21, they were culled humanely and splenocytes and serum were harvested. Samples of liver and spleen were also taken for histological analysis. This was repeated in an independent study, in 6-weeks old A129 mice only (n = 3 mice per group).

Using histological examination by HE staining, lesions in liver and spleen were not detected in vaccinated A129 or 129Sv/Ev animals at day 21. Therefore no detectable pathology was caused by the vaccine itself.

**Figure 3:**
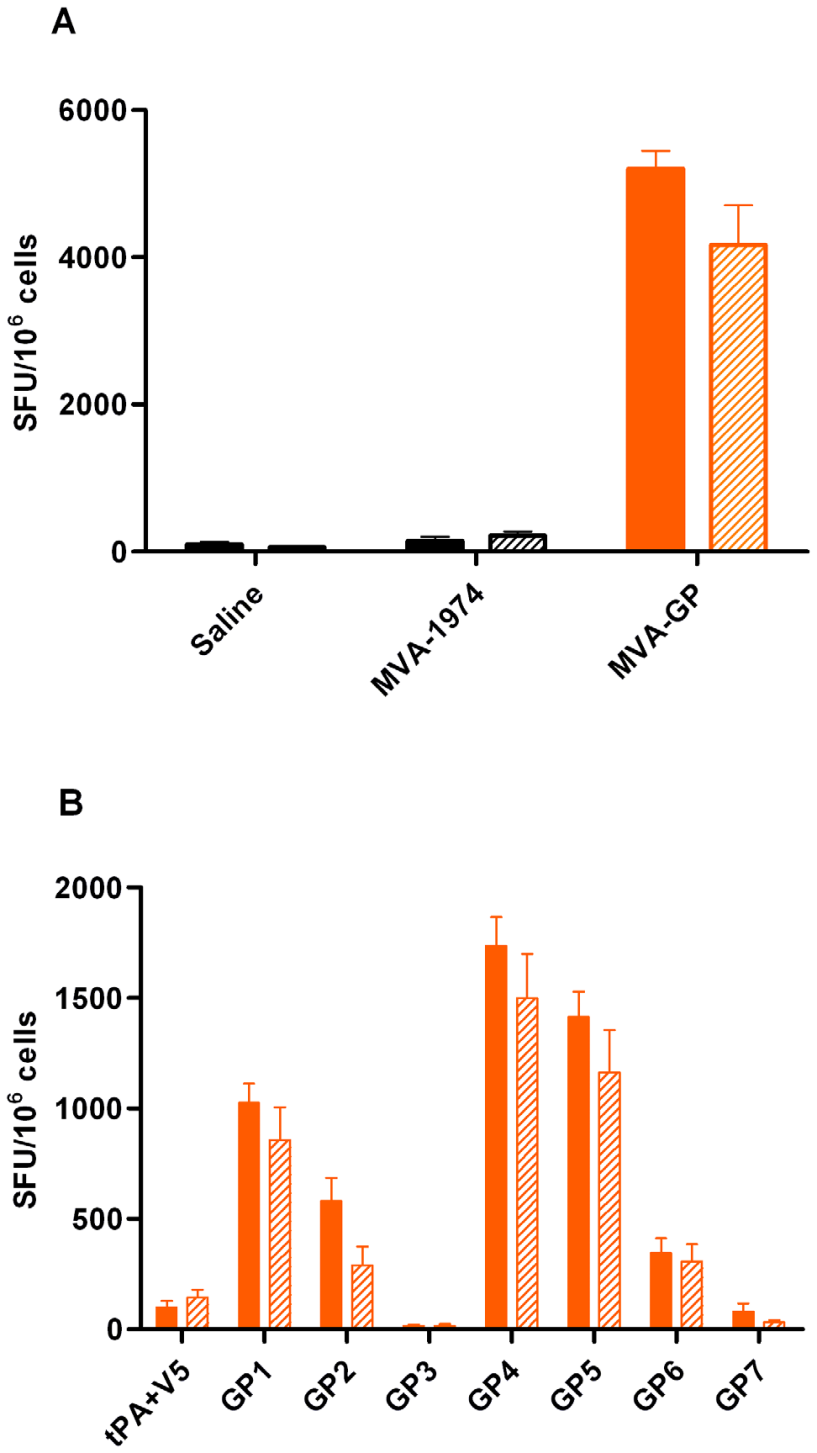
IFN-γ ELISpot responses from vaccinated A129 and 129Sv/Ev mice, 7 days after booster vaccination. Splenocytes from animals vaccinated with MVA-GP (orange), MVA 1974 or saline (black) were restimulated with peptides derived from the CCHFv glycoprotein. A129 mice data (solid bars) were pooled from 2 independent experiments (n = 8). 129Sv/Ev mice n = 5 (hatched bars). Mean ± SEM is plotted. (A) Summed antigen responses from all peptide pools. Splenocytes from MVA-GP vaccinated mice, but not control mice, responded to GP-specific peptides, indicating similar T-cell responses between mouse strains. (B) Antigen responses from MVA-GP vaccinated mice, according to peptide pool. Immunogenicity was not evenly distributed across the antigen, but some peptide pools were more immunogenic than others. Responses were specific to the glycoprotein, and similar between mouse strains.

IFN-γ ELISPOT results from splenocytes prepared on day 21 showed that MVA-GP immunised mice generated cellular immune responses specific to the CCHFv glycoprotein (Fig 3A). When the responses to viral glycoprotein were further subdivided into 7 separate peptide pools, the same pools were optimal between mouse strains, whereas other peptide pools were consistently non-immunogenic. The peptide pools inducing the highest ELISPOT responses, GP4, GP5, GP1 and GP2, mapped largely to the NSm, N-terminus of G_C_, mucin-like and GP38 domains, respectively. The majority of the G_N_ protein, except for 57 C-terminal residues which fell into peptide pool GP4, mapped to pool GP3 and did not induce a specific T-cell response. The central and C-terminal regions of G_C_, which mapped to peptide pools GP6 and GP7, also failed to stimulate T cells. Interestingly, the non-structural domains therefore contained highly immunogenic T-cell epitopes.

No statistical difference in immune responses was observed between strains of mice after MVA-GP vaccination, showing no effect of abrogated type I IFN receptors on T-cell induced vaccine responses. T-cell responses to peptides derived from the tPA or V5 regions, or from an irrelevant antigen (CCHFv nucleoprotein) were negligible, indicating specificity of the response (Fig 3B).

**Figure 4:**
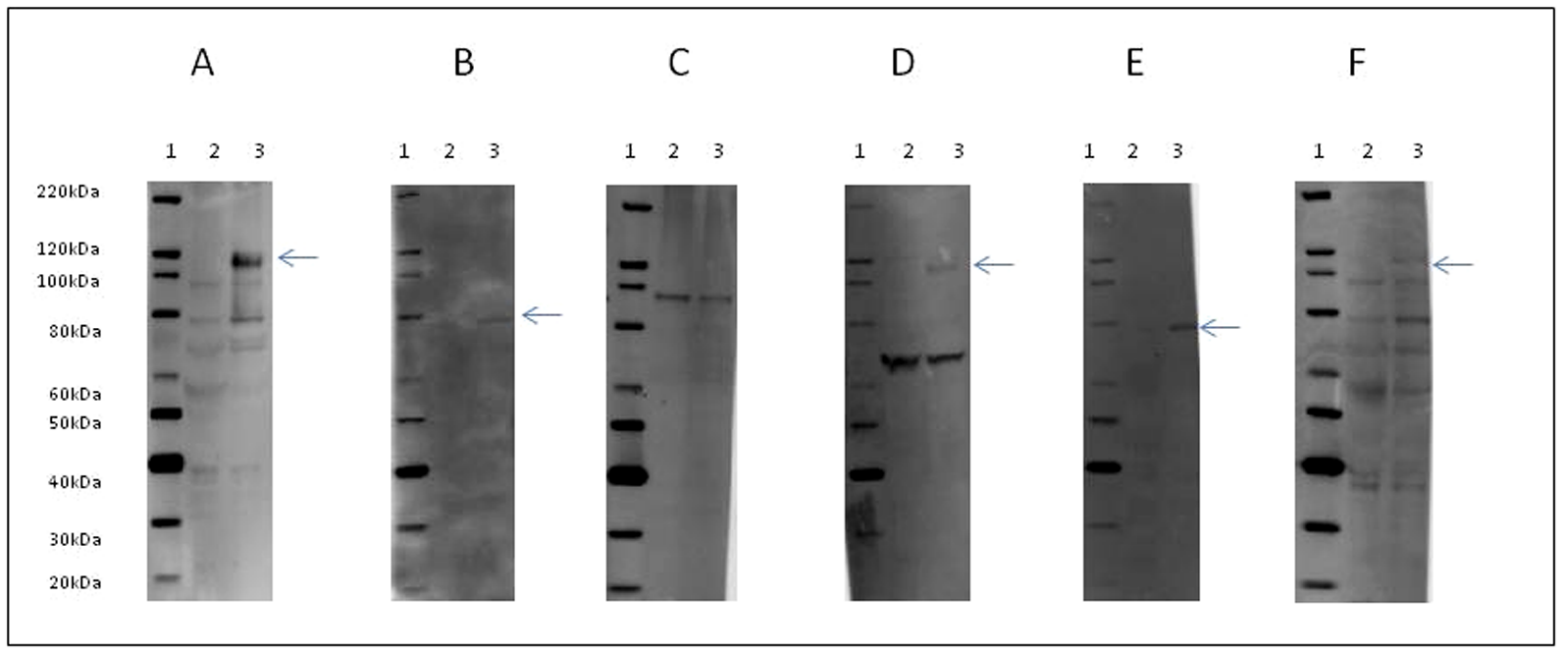
Antibody responses from vaccinated A129 and 129Sv/Ev mice. Sera from vaccinated mice were tested for reactivity with CCHFv-infected (lane 3) or uninfected (lane 2) SW13 cells by Western blotting. Lane 1 shows a molecular weight marker. Blots show proteins reactive with serum from representative individual animals 7 days after booster vaccination (A–E) or representative pooled serum 14 days after booster vaccination (F). Secondary antibody used was specific for mouse IgG (A–C, F), or mouse IgG, IgA and IgM (D–E). Arrows highlight CCHFv-specific proteins, indicating specific antibody responses in both mouse strains. (A) 129Sv/Ev mouse vaccinated with MVA-GP. (B, D–E) A129 mouse vaccinated with MVA-GP. (C) A129 mouse vaccinated with MVA 1974. (F) Pooled sera from A129 mice vaccinated with MVA-GP.

These vaccinated animals were also tested for induction of a CCHFv-specific humoral response by MVA-GP, using Western blotting. IgG antibody reacting with a protein of approximately 114 kDa was detected in 3 out of 5 vaccinated animals' sera from 129Sv/Ev mice collected on day 21 of the vaccination schedule (Fig 4A). In A129 mice, a CCHFv-specific IgG antibody response was detectable by Western blot in only 1 out of 8 individual animals, which recognised a 79 kDa protein (Fig 4B). A randomly selected A129 mouse that received the MVA 1974 negative control was tested, and no CCHFv-specific antibody response was seen (Fig 4C).

Sera from the 8 A129 animals vaccinated with MVA-GP were also assessed by Western blot for an early phase immune response using a detector antibody specific for mouse IgG, IgA and IgM. Antibodies specific for a 79 kDa CCHFv protein were detected in the same animal that had tested positive with anti-IgG only (Fig 4E). Since the pattern of protein recognition in this individual was similar in both assays (i.e. Figs 4B and 4E), it can be inferred that the antibodies that recognised the 79 kDa protein were predominantly IgG. Broadening the sensitivity to additional antibody classes in this way, detected antibodies specific for a protein of approximately 114 kDa (Fig 4D) in a further 4 animals. In these individuals, therefore, IgM was probably the predominant antibody class since IgA is not expected in serum. Sera from the remaining 3 A129 mice did not recognise any CCHFv-specific proteins (data not shown).

In a separate study, 5–6 weeks old A129 mice were vaccinated intramuscularly with 10^7^ pfu/animal MVA-GP or MVA 1974 (25 mice per group) at day 0. Animals received a booster vaccination at day 14. At day 28, sera were collected, heat inactivated and pooled. Each pool contained sera from 5–6 animals. In 4 out of 5 pools from MVA-GP vaccinated animals, an IgG antibody response to the protein of approximately 114 kDa was detected (Fig 4F). The remaining pool could not be adequately assessed due to high background staining, most likely caused by severe haemolysis in this pool. One of the serum pools from the MVA 1974 vaccinated control group was tested for IgG antibodies, and no reactivity was observed.

Collectively, this evidence indicated that A129 mice mounted humoral responses that recognised similar antigens to the 129Sv/Ev immunocompetent mice, but the kinetics were delayed. By day 21 of the vaccination protocol (7 days post-boost), most 129Sv/Ev animals had a detectable IgG response. In contrast, most A129 animals underwent class switching from IgM to IgG between days 21–28.

**Figure 5:**
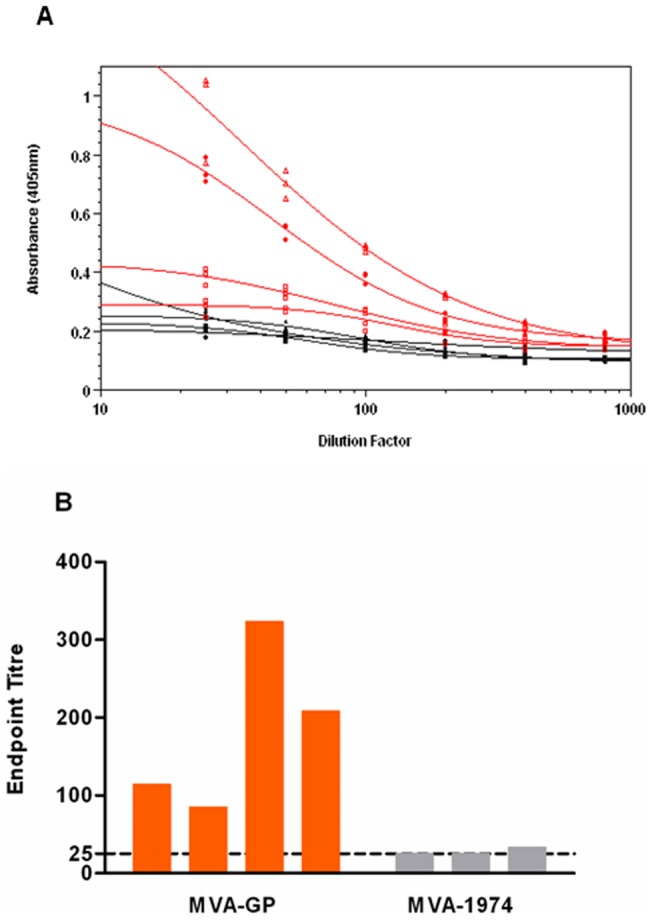
ELISA analysis of antibodies from A129 mice, 14 days after booster vaccination. Pooled sera from A129 mice, 14 days after booster vaccination with MVA-GP (orange) or MVA 1974 (grey), were tested by ELISA for IgG, IgA or IgM antibodies specific for G_N_. Normal mouse serum (black) was a negative control. (A) Absorbance of diluted samples, and curve fit, from which endpoint titres were calculated where y = 0.250. Each point represents one of triplicate values for each pool. (B) Endpoint titres for each pool tested. The lower limit of detection was 25. All four pools from MVA-GP vaccinated mice were positive. One out of three pools from MVA 1974 vaccinated mice was very weakly positive; the remaining two were negative. Therefore, only the MVA-GP vaccine induced significant G_N_-specific antibodies, not the MVA 1974 negative control.

To further investigate the antibody response, the serum pools from day 28 were also tested by indirect ELISA against the G_N_ ectodomain, in an assay specific for IgG, IgA or IgM. Severely haemolysed pools were excluded from analysis due to non-specific reactivity. To show true differences between samples, an absorbance endpoint value of 0.250 was selected manually, where the curves were the most parallel (Fig 5A). The lower limit of detection for the assay was an endpoint titre of 25, the lowest dilution factor used. Four pools from mice vaccinated with MVA-GP were tested, which reached endpoint titres of 114, 85, 323 and 208. One of the pools from mice vaccinated with MVA 1974 was weakly positive, with an endpoint titre of 33. The remaining 2 pools tested were below the lower limit of detection (Fig 5B). Therefore, the MVA-GP vaccine specifically induced antibodies that recognised G_N_.

Although all MVA-GP vaccinated pools were positive for CCHFv-specific antibodies, responses were notably higher in 2 of them. This might have been due to sera from strongly responding animals being pooled together, or due to the presence of serum from non-responders in the lower titre pools. When the assay was repeated, using a conjugate specific for IgM only, all samples were negative (data not shown). As IgA is not expected in serum, we conclude that all positive A129 samples therefore contained predominantly IgG at day 28.

### Efficacy of MVA-GP in CCHF-susceptible mice

To establish the lethal dose of CCHFv, virus diluted in PBS was intradermally inoculated into 5-7 weeks old A129 mice. Concentrations of 1000, 100, 10 and 1 TCID_50_ (5 animals per group) demonstrated that 100 TCID_50_ was the lowest dose that caused 100% of infected A129 mice to reach humane clinical endpoints.

Six-week old A129 mice (9 per group) were vaccinated with MVA-GP or MVA 1974 at 10^7^ pfu/animal, or an equivalent volume of saline, on day 0. All animals received a booster vaccination at day 14. On day 28, animals were challenged with 200 TCID_50_ CCHFv intradermally (i.e. double the minimum lethal dose). Four days after challenge (day 32), 3 randomly selected animals from each group were sacrificed for histological examination and viral load testing. At 14 days post-challenge (day 42), all remaining animals were killed humanely and samples collected for histology and viral load testing.

**Figure 6:**
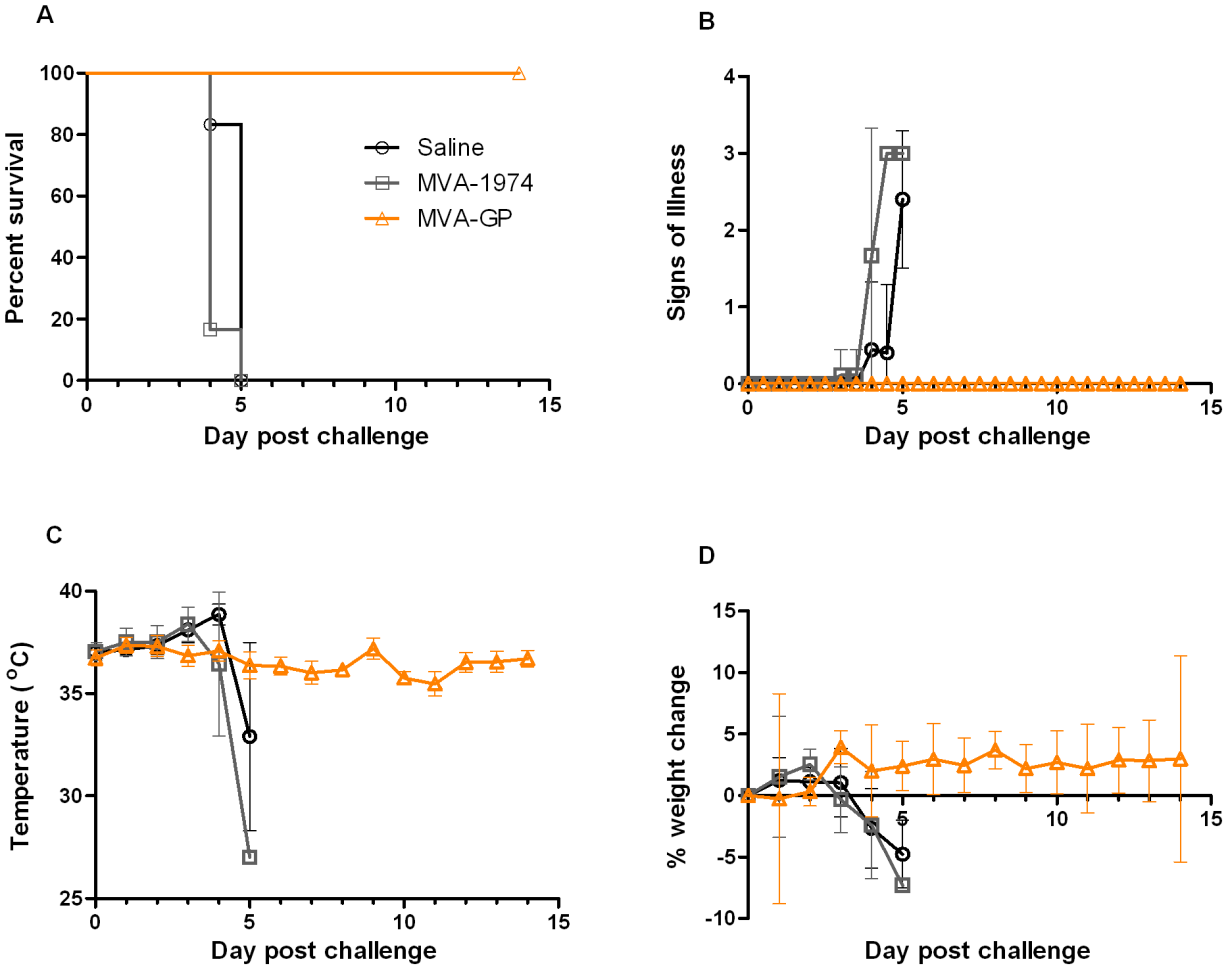
Efficacy of MVA-GP in A129 mice challenged with CCHFv. A129 mice (n = 6) were challenged with double the minimum lethal dose of CCHFv 14 days after booster vaccination with MVA-GP (orange triangles), MVA 1974 (grey squares) or saline (black circles) and monitored for the following 14 days. All mice vaccinated with MVA-GP survived lethal challenge, and did not show any signs of illness, become febrile, or exhibit weight loss. (A) Percentage of surviving animals. (B) Signs of illness. Mean ± standard deviation is plotted. (C) Body temperature. Mean ± standard deviation is plotted. (D) Weight change as a percentage of body weight at challenge. Mean ± standard deviation is plotted.

Animals in the control groups began to show clinical signs 3 days after challenge. These increased in severity and all animals were euthanised 4–5 days post-challenge (Fig 6A & B). In contrast, all animals that received the MVA-GP vaccination had no clinical signs up to 14 days post-challenge (day 42). The study was stopped at this point as vaccinated animals had survived more than 3 times longer than control groups, and were considered to have passed beyond the critical phase of disease. Animals that were not protected from disease exhibited an initial rise in body temperature, followed by a sharp reduction as they succumbed. In contrast, the temperature of MVA-GP vaccinated animals remained stable throughout the study (Fig 6C). Control animals lost 5–10% of their original body weight within 3–5 days of challenge, whereas the weights of the MVA-GP vaccinated mice remained stable throughout the study (Fig 6D).

### Histopathological findings in vaccinated, CCHFv challenged mice

**Figure 7:**
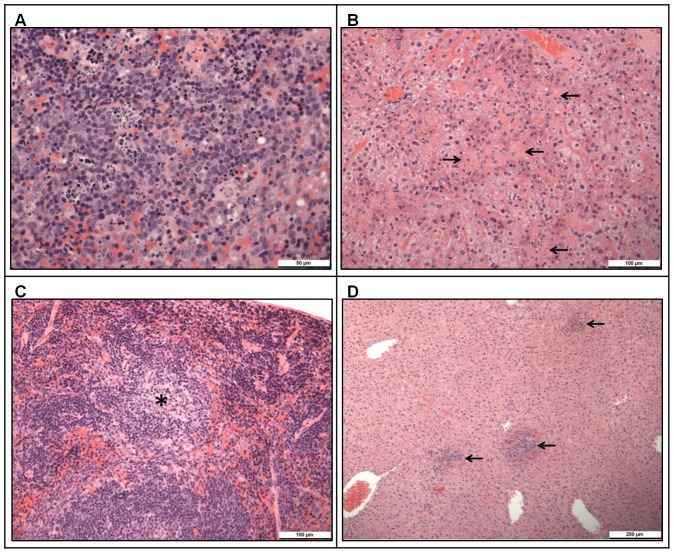
Tissue histology of A129 mice, 4 days after challenge with CCHFv. A129 mice were challenged with double the minimum lethal dose of CCHFv, 14 days after booster vaccination with MVA 1974 (A–B) or MVA-GP (C–D). Four days after challenge, sections of spleen (A, C) and liver (B, D) were fixed, HE stained, and examined for pathology. More severe pathology was found in mice that received MVA 1974, compared to those that received MVA-GP. (A) Marked lymphocyte loss with prominent apoptotic bodies, and infiltration by macrophages. (B) Marked, multifocally extensive hepatocyte necrosis (arrows). (C) A single infiltration of macrophages in the white pulp (asterisk) (scored minimal). (D) Scattered, multifocal areas of hepatocellular necrosis with a mixed inflammatory cell infiltrate (arrows) (scored moderate).

The liver of one animal that received MVA 1974 prior to CCHFv challenge appeared normal at day 32 of the vaccination schedule. All other challenged animals in the MVA 1974 and saline groups showed lesions in both the spleen and liver (Table 1). In the spleen, microscopic changes comprised patchy to diffuse infiltration of the parenchyma by macrophages, primarily involving the red pulp, with varying degrees of effacement of white pulp (Fig 7A). The latter involved lymphocyte loss and apoptosis, and was characterised by tingible body macrophages and apoptotic bodies. In the liver, changes comprised multifocal to diffuse, hepatocyte necrosis, characterised by cytoplasmic eosinophilia and nuclear pyknosis and accompanied frequently with a mixed inflammatory cell infiltration, mainly of polymorphonuclear leukocytes (Fig 7B).

**Table 1:** Severity of microscopic lesions in HE stained tissues from vaccinated A129 mice, challenged with CCHFv.

	Severity	Group			
		Saline (day 32–33)	MVA 1974 (day 32–33)	MVA-GP (day 32)	MVA-GP (day 42)
Spleen	Normal	0	0	1	6
	Minimal	1	1	2	0
	Mild	2	3	0	0
	Moderate	2	1	0	0
	Marked	4	4	0	0
Liver	Normal	0	1	2	6
	Minimal	0	0	0	0
	Mild	2	0	0	0
	Moderate	3	3	1	0
	Marked	4	5	0	0

Numbers of animals in each group, according to severity rating of histological lesions.

In the MVA-GP group, at day 32, in the spleen, minimal changes were seen in two out of three animals (Fig 7C). In one of these animals, moderate changes were identified in the liver, characterised by multifocal changes of hepatocyte necrosis and mixed inflammatory cell infiltration (Fig 7D). In the remaining six animals in this group, which survived to day 42, lesions were not identified.

**Figure 8:**
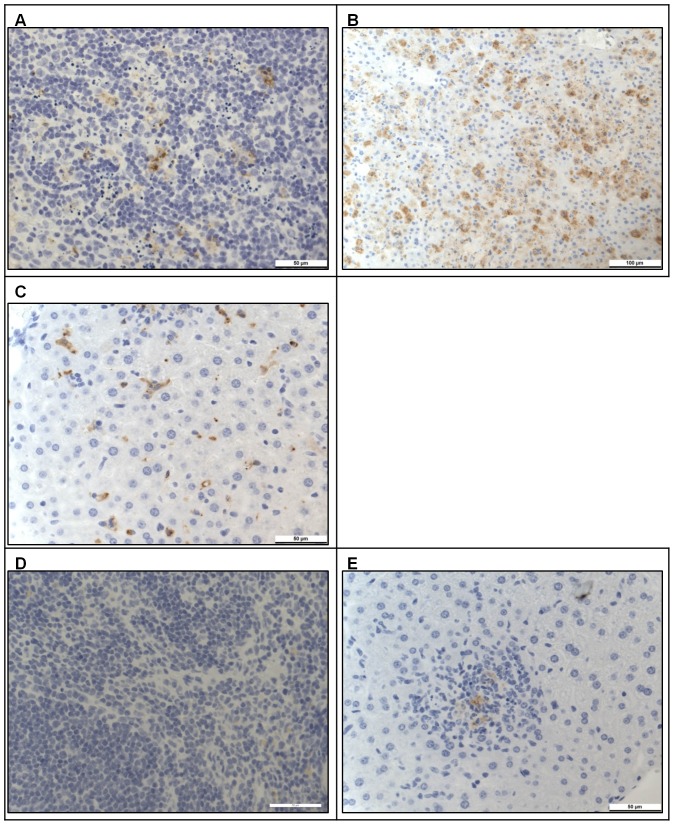
Immunohistochemistry of tissues from A129 mice, 4 days after challenge with CCHFv. A129 mice were challenged with double the minimum lethal dose of CCHFv, 14 days after booster vaccination with MVA 1974 (A–C) or MVA-GP (D–E). Four days after challenge, sections of spleen (A, D) and liver (B–C, E) were fixed, immunohistochemically stained with CCHFv-specific antibody, and examined microscopically. Tissues in panels A, B, D and E were from the same individuals as shown in Figures 7A, 7B, 7C and 7D, respectively. A diffuse staining pattern of viral proteins was found in tissues from animals that received the MVA 1974 negative control. However, in MVA-GP vaccinated animals, the only staining found was of a minimal degree, in liver from one individual. (A) A few, scattered cells with cytoplasmic staining within the parenchyma. (B) Frequent, diffuse, positively stained hepatocytes. (C) Scattered, small, elongated cells consistent with Kupffer cells, with cytoplasmic staining. (D) Normal parenchyma. (E) A few, positively stained cells within an inflammatory cell focus.

Selected samples were also examined by immunohistochemistry (Table 2). In all MVA 1974 or saline-treated animals, positive staining was present in both spleen and liver. This included positive staining in two animals vaccinated with MVA 1974, which did not exhibit clinical signs. In the spleen, the majority of positive cells appeared to be macrophages in the red pulp, with only scattered cells in the white pulp (Fig 8A). Liver samples from animals with clinical signs had diffuse, positive cytoplasmic staining of hepatocytes (Fig 8B). Two animals vaccinated with MVA 1974 which did not show clinical signs when killed humanely at day 32, had positive staining predominantly in the Kupffer cells (macrophages lining the sinusoids) (Fig 8C). By contrast, in the MVA-GP group, the spleens from 4/4 animals examined (3 at day 32 and 1 at day 42) did not show staining (Fig 8D). In the livers of these animals, staining was positive in 1 from day 32, while the remaining 3 animals were negative. Occasional hepatocytes in scattered, inflammatory foci, showed positive staining (Fig 8E), indicating an inflammatory response to viral antigen.

**Table 2:** Frequency of immunohistochemically stained cells in tissues from selected vaccinated A129 mice, challenged with CCHFv.

	Frequency	Group			
		Saline (day 32–33)	MVA 1974 (day 32–33)	MVA-GP (day 32)	MVA-GP (day 42)
Spleen	Normal	0	0	3	1
	Minimal	4	4	0	0
	Moderate	2	3	0	0
	Marked	1	0	0	0
Liver	Normal	0	0	2	1
	Minimal	1	2	1	0
	Moderate	3	1	0	0
	Marked	3	4	0	0

Numbers of animals in each group, according to frequency of cells stained by immunohistochemistry.

### Viral load analysis

**Figure 9:**
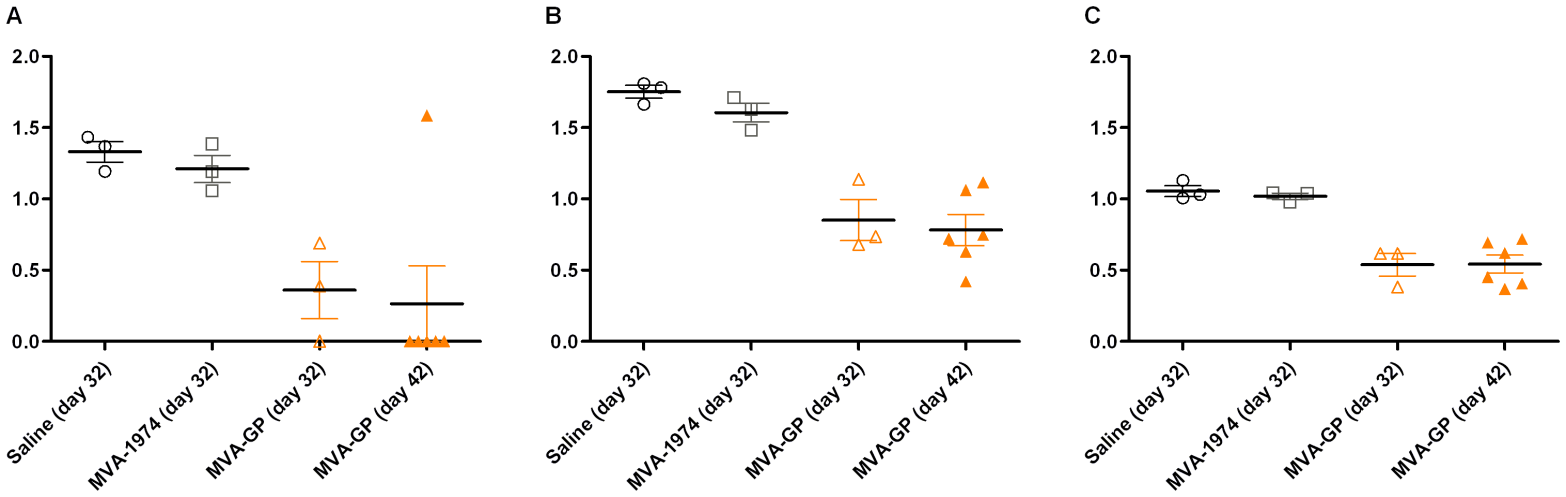
Normalised viral load analysis of CCHFv RNA by RT-PCR. A129 mice (n = 9) were challenged with double the minimum lethal dose of CCHFv, 14 days after booster vaccination with MVA-GP, MVA 1974, or saline. Four days post-challenge (day 32), 3 randomly selected animals from each group were killed humanely and analysed by RT-PCR for CCHFv gene expression, normalised to mouse HPRT gene expression. Fourteen days post-challenge (day 42), all surviving animals were killed humanely and also analysed. Each point represents the mean value of triplicate measurements in an individual animal. Lines show mean ± SEM. In all tissues tested, viral load was significantly lower in MVA-GP vaccinated mice than in control groups. Within the MVA-GP group, there was no significant difference in viral load between 4 days and 14 days post-challenge. (A) Blood. (B) Spleen. (C) Liver.

Viral load was analysed by RT-PCR of CCHFv S segment in blood, spleen, and liver from 3 animals per group at day 32 of the vaccination schedule, and from all surviving animals at day 42. CCHFv expression was normalised to expression of the HPRT reference gene (Fig 9). In the control groups at day 32, viral RNA was detected in all tissues tested, with no statistically significant difference between saline and MVA 1974. In the MVA-GP vaccinated group at day 32, viral RNA was detected in the blood of only two out of three animals, although all animals had detectable viral RNA in the spleen and liver. Normalised CCHFv expression was significantly lower in MVA-GP vaccinated animals compared to control groups in all tissues (p = 0.05). At day 42, viral RNA was detectable in the blood of only 1 out of 5 vaccinated mice, but was detected in all other tissues tested. There was no statistically significant difference between day 32 and day 42 samples from MVA-GP vaccinated animals.

To investigate whether infectious virus was still present at the end of the study, homogenised supernatants of spleen and liver tissue from 5 of the 6 survivors to day 42 (including the viraemic individual), were incubated under viral culture conditions for 48 hours. Although cytopathic effects are usually observed at this timepoint, and the positive control isolate cultured in parallel induced extensive cytopathicity, none of the mouse survivor samples induced any observable cytopathic effects. It is possible that a longer culture period may have been necessary to observe cytopathicity, but these preliminary results suggest that the RNA detected at day 42 was unlikely to represent viable virus.

## Discussion

We have developed an MVA-vectored candidate vaccine against CCHF, based on the full-length glycoprotein precursor encoded by the CCHFv M segment. MVA has a proven safety record, having been used in the latter stages of the smallpox eradication campaign [39]. MVA can be inexpensively manufactured to GMP and has an established regulatory package for development as an Investigational New Drug. The proven clinical safety record, induction of humoral and cellular antigen-specific immune responses, and thermostability [40] for application in remote regions without an established cold chain make MVA a promising viral vector to deliver a potential CCHF vaccine. Indeed, MVA vaccines are currently in clinical trials up to phase III for diseases including tuberculosis, malaria, HIV-AIDS, cancer, influenza, and Hepatitis C [41]–[47].

Glycoproteins are often targeted in novel vaccine designs, even for highly variable pathogens such as HIV-1 and influenza virus (see [48] for a review) since their exposed position on the virion surface makes them accessible for antibody binding and neutralisation.

The CCHF glycoprotein precursor undergoes co- and post-translational proteolytic cleavage events in the endoplasmic reticulum and Golgi body, at the conserved motifs RSKR, RRLL and RKPL [15], [38], [49]. This complex series of maturation steps can cause difficulties when attempting to express recombinant G_N_ or G_C_ *in vitro*. The use of a viral vector delivery system bypasses these problems and is more likely to lead to authentic protein expression patterns. As G_N_, but not G_C_, contains a Golgi localisation signal, interaction between the two glycoproteins is required for G_C_ to travel to the Golgi [17], [50]. Therefore, although G_C_ may contain more neutralising or protective epitopes [17], [51], the inclusion of G_N_ in the vaccine design may have an advantageous effect, by assisting correct trafficking.

In this work, the vaccine candidate encoded the non-structural accessory domains (mucin like domain, GP38 and NSm), as well as G_N_ and G_C_. This is similar to a candidate DNA vaccine described by Spik *et al* [19], but in contrast to an oral vaccine that expressed G_N_ and G_C_ only [20]. The structural proteins rely on N-glycosylation of G_N_ for their correct folding and transport [52], and G_N_ in turn has a complex maturation process involving sequential cleavage from the accessory domains [14], [15], [38], [49]. The mucin-like and GP38 domains are required for correct transport of G_N_ to the Golgi apparatus [17]. There is a complex interplay between the products of the glycoprotein precursor, and the absence of a critical component could have had an unexpected effect on processing and expression of the antigens.

In the MVA-GP vaccine, the full-length glycoprotein open reading frame was controlled by the mH5 poxvirus promoter [33], for increased stability [53], and strong early expression to drive a cytotoxic T-lymphocyte response. A leader sequence from human tPA was added for increased immunogenicity and intracellular transport [36], [37]. V5 was added for identification of expressed protein by immunolabelling.

An intradermal challenge route was chosen for our CCHFv infection model since this closely resembles the major natural route of infection via tick bite. The incubation period of CCHF in humans generally varies between 1-7 days [4], [6], but can be as long as 22 days [54]. A shorter incubation period has been associated with acquiring infection by tick bite, compared to by contact with infected human blood [55], and in nosocomial transmission, a shorter incubation period was associated with fatal outcome [54].

Similar studies with other tick-borne pathogens have also modelled infection and disease using the intradermal route to experimentally mimic tick bite including those using *Ehrlichia* [56], Lyme Disease [57] and *Rickettsia* [58]. Previous characterisations of CCHFv infection in mouse models have focused on the intraperitoneal route, although a recent paper has also characterised other inoculation routes, including subcutaneous [11].

The time to onset of clinical signs, and the survival time in unprotected groups seen in this study, were consistent with those seen by Zivcec *et al* [11] with a challenge dose of 100 TCID_50_ delivered subcutaneously. However, the lethal dose observed by that group was 0.05 TCID_50_, compared to >10 TCID_50_ in the present study. This difference could be explained by the inoculation route used (subcutaneous versus intradermal), or the genetic backgrounds of the mice used (C57Bl/6 versus 129Sv/Ev). Histopathological lesions seen in the liver and spleen, by both HE staining and immunohistochemistry, were consistent with previous observations [11], [12].

In this work, both cell-mediated and humoral immune responses were assessed in mouse models, with or without fully functional innate immunity. Immunogenicity data from additional MHC genetic backgrounds would be interesting, but would be of limited value without the ability to follow up with efficacy studies.

Characterising immune responses in convalescent patients could illustrate the type of protective immunity required of an effective vaccine. However, very few clinical studies have examined cellular immune responses to CCHF. Akinci *et al* did not report antibody levels, but found significantly higher levels of CD3^+^ CD8^+^ T cells in fatal cases compared to non-fatal cases, which correlated with viral load [59]. Therefore CCHF induces proliferation of cytotoxic T-cells, but these are incapable of controlling infection in fatal cases. Another report also found elevated levels of CD3^+^ CD8^+^ cells in paediatric CCHF cases, but there were no fatal outcomes in this study group for comparison [60].

In order to efficiently capture both CD4^+^ and CD8^+^ immune responses to the MVA-GP vaccine, peptides used in the ELISPOT assay were 20 amino acid residues in length. This approach has been shown to stimulate both T cell subsets [61]. A positive T-cell response was only seen in the group that survived lethal challenge. As immunogenicity studies required terminal sacrifice, however, analysis of pre-challenge immune responses and survival studies occurred in different individuals. Any correlation could therefore only be applied to the group as a whole.

The reason for the variable T-cell immunogenicity observed across the glycoprotein domains is unclear, but it is encouraging that the highest levels were seen in the more conserved domains. The mucin-like domain is highly variable across CCHFv strains, with up to 56.4% difference in amino-acid identity, compared to up to 8.4% difference for the rest of the glycoprotein open reading frame [14]. Although the relevance of the cellular response to protection is still unknown, this is promising for designing a vaccine that confers cross-strain protection.

Most clinical CCHF immunity studies have examined serum antibody levels against the nucleoprotein [62], [63]. Patients with a fatal outcome have a weak or absent antibody response, and Ozturk *et al* found significant differences between IgM and IgG levels in fatal vs. non-fatal cases [64]. However, other studies were unable to find a significant relationship between the presence of IgM and IgG antibodies and clinical outcome, but found that viral load was a better predictor of prognosis [65], [66]. Virus titres decreased in survivors during the first week of disease, independently of antibodies, suggesting a critical role of innate or cellular immune mechanisms [66].

Monoclonal antibodies, raised against viral glycoproteins, identified protective epitopes in both G_N_ and G_C_. However, only antibodies recognising epitopes in G_C_ were neutralising *in vitro* [17]. Therefore direct neutralisation is not the only mechanism by which antibodies can protect against CCHF. Protective antibodies were also found to be cross-reactive across CCHFv clades [51], which is again encouraging for designing a vaccine that confers cross-strain protection.

In this report, we found antibody recognition of similar CCHFv glycoproteins in both the wild-type 129Sv/Ev and A129 mouse strains. The response in A129 mice, however, was slightly delayed. On day 21 of the vaccination schedule, most 129Sv/Ev animals had a detectable IgG response. However, at this time point, most of the A129 humoral response was only detected when the assay specificity was broadened to include IgM. This response was unlikely to be transient, since a predominantly IgG response was detected after an additional 7 days. If protection is mediated via the humoral response, IgG will be required for long-term immune memory, whereas IgM is likely to provide only short term protection from disease. Although IgG was observed at day 28 (the time of challenge) in A129 mice, a longer interval between vaccination and challenge will be needed to confirm that the vaccine induces immune memory.

Quantitation by ELISA also found varying levels of specific antibodies in pooled sera from approximately 20 vaccinated A129 mice, 14 days after the booster was administered. Unfortunately it was not possible to test individual sera from this time point to quantitate the range of individual responses (samples were not available prior to pooling). Since CCHFv-infected cell lysate could not be validated for ELISA, recombinant G_N_ ectodomain was used as the capture antigen. Therefore, vaccine-induced antibodies specific for other domains would not have been detected by this assay. Specific antibodies were only seen in the group that survived lethal challenge, but not all individuals had a detectable response, using either of the methods described. Therefore a direct correlation could not be drawn between induction of CCHFv-specific antibodies, and protection.

At 4 days post-challenge, randomly selected animals were sacrificed for viral load analysis and histological examination. These included 2 animals from the MVA 1974-treated group that did not display any clinical signs of illness. Immunohistochemistry on liver samples of these animals detected viral antigen in the Kupffer cells. An association with Kupffer cells has also been noted in a STAT-1 knockout model of CCHFv, and in human cases [11], [12]. Immunohistochemistry was therefore able to distinguish between MVA-GP vaccinated, and control vaccinated individuals in the absence of clinical signs.

It is probable that the MVA-GP vaccinated animals were not completely free from infection, but developed a subclinical disease, as viral RNA was detected in all animals at four days post-challenge. A positive correlation between viral load and severity of disease has been observed in patients [65], [66], consistent with our observations in the mouse model.

At the end of the efficacy study, viral RNA was still detected in surviving animals, although not all individuals had detectable viraemia. Although the consistent levels of viral load seen between day 32 and day 42 may have indicated ongoing, subclinical viral replication, it is likely that the RNA was released from defective viral particles, or from virus that had been inactivated by effective immunity e.g. neutralising antibodies. Initial attempts to amplify live virus from day 42 tissue samples were unsuccessful; therefore the possibility that this vaccine induced sterilising immunity has not been excluded. Interestingly, prolonged viraemia assessed by RT-PCR has also been observed in convalescent patients, but that study did not report any attempts to recover viable virus [67].

The MVA-GP vaccine induced comprehensive cellular and humoral immunity to CCHFv, which translated into protection against severe disease, such that no clinical signs of illness were apparent during the study. Furthermore, the adaptive immune response elicited by the vaccine was not substantially affected by the absence of the type I IFN receptor in the A129 mice. Protection correlated with T-cell responses, but not antibody induction, although further studies are required to determine exactly which is the protective component. Further directions for this work could also include testing the longevity of demonstrated protective effects, and investigating whether MVA-GP confers protection against multiple CCHFv strains.

This report provides the first demonstration of protection by a CCHF candidate vaccine in a lethal animal challenge model, and validates this application of the IFNα/βR knockout adult mouse model. The MVA-based vaccine approach gives promise that a modern CCHF vaccine, that can meet international regulatory approval, is possible. Currently, the data look extremely promising and represent major progress in the search for a medical intervention that can be used to protect against CCHF.

## Supporting Information

File S1 Sequence of Insert. The full nucleotide sequence of the inserted cassette used to generate MVA-GP. The translation of the tPA-GP-V5 fusion protein is also given.
(DOCX)
