# Supplementary material for: A Novel Vaccine against Crimean-Congo Haemorrhagic Fever Protects 100% of Animals against Lethal Challenge in a Mouse Model
Source: PLoS One. 2014 Mar 12;9(3):e91516. doi: 10.1371/journal.pone.0091516 (PMC3951450; doi:10.1371/journal.pone.0091516)
Supplement: File S1 — Sequence of Insert. The full nucleotide sequence of the inserted cassette used to generate MVA-GP. The translation of the tPA-GP-V5 fusion protein is also given. (DOCX) [file pone.0091516.s001.docx]

6171 bp linear DNA

Nucleotide Sequence

1 GTACCAGGCG CGCCTTTCAT TTTGTTTTTT TCTATGCTAT AAATGGTGAG CAAGGGCGAG

61 GAGCTGTTCA CCGGGGTGGT GCCCATCCTG GTCGAGCTGG ACGGCGACGT AAACGGCCAC

121 AAGTTCAGCG TGTCCGGCGA GGGCGAGGGC GATGCCACCT ACGGCAAGCT GACCCTGAAG

181 TTCATCTGCA CCACCGGCAA GCTGCCCGTG CCCTGGCCCA CCCTCGTGAC CACCCTGACC

241 TACGGCGTGC AGTGCTTCAG CCGCTACCCC GACCACATGA AGCAGCACGA CTTCTTCAAG

301 TCCGCCATGC CCGAAGGCTA CGTCCAGGAG CGCACCATCT TCTTCAAGGA CGACGGCAAC

361 TACAAGACCC GCGCCGAGGT GAAGTTCGAG GGCGACACCC TGGTGAACCG CATCGAGCTG

421 AAGGGCATCG ACTTCAAGGA GGACGGCAAC ATCCTGGGGC ACAAGCTGGA GTACAACTAC

481 AACAGCCACA ACGTCTATAT CATGGCCGAC AAGCAGAAGA ACGGCATCAA GGTGAACTTC

541 AAGATCCGCC ACAACATCGA GGACGGCAGC GTGCAGCTCG CCGACCACTA CCAGCAGAAC

601 ACCCCCATCG GCGACGGCCC CGTGCTGCTG CCCGACAACC ACTACCTGAG CACCCAGTCC

661 GCCCTGAGCA AAGACCCCAA CGAGAAGCGC GATCACATGG TCCTGCTGGA GTTCGTGACC

721 GCCGCCGGGA TCACTCTCGG CATGCACGAG CTGTACAAGT AAGCGGCCGC TGGTACCCAA

781 CCTAAAAATT GAAAATAAAT ACAAAGGTTC TTGAGGGTTG TGTTAAATTG AAAGCGAGAA

841 ATAATCATAA ATAAGCccgg tGCCACCATG gatgcaatga agagagggct ctgctgtgtg

901 ctgctgctgt gtggagcagt cttcgtttcg cccagccagg aaatccatgc ccgattcaga

961 agaggagcca gatctcccAT CAAACAAGTT TGTACAAAAA AGCAGGCTca tatatcatta

1021 atgtatgcaa tcctttgcct acagctgtgt ggtctgggag agactcatgg atcacacaat

1081 gaaactagac acaataaaac agacaccatg acaacacacg gtgataaccc gagctctgaa

1141 ccgccagtga gcacggcctt gtctattaca cttgacccct ccactgtcac acccacaaca

1201 ccagccagtg gattagaagg ctcaggggaa gtctacacat cccctccgat caccaccggg

1261 agcttgcccc tgtcggagac aacaccagaa ctccctgtta caaccggcac agacacctta

1321 agcgcaggtg atgtcgatcc cagcacgcag acagccggag gcacctccgc accaacagtc

1381 cgcacaagtc tacccaacag ccctagcaca ccatctacac cacaagacac acaccatcct

1441 gtgagaaatc tactttcagt cacgagtcct gggccagatg aaacatcaac accctcggga

1501 acaggcaaag agagctcagc aaccagtagc cctcatccag tctccaacag accaccaacc

1561 cctcctgcaa cagcccaggg acccactgaa aatgacagtc acaacgccac tgaacaccct

1621 gagtccctga cacagtcagc aaccccaggc ctaatgacct ctccaacaca gatagtccac

1681 ccacaaagtg ccacccccat aaccgttcaa gacacacatc ccagtccaac gaacaggtct

1741 aaaagaaacc ttaagatgga aataatcttg actttatctc agggtttaaa aaagtactat

1801 gggaaaatat taaggcttct gcaactcacc ttagaggagg acactgaagg tctactggaa

1861 tggtgtaaga gaaatcttgg tcttgattgt gatgacactt tctttcaaaa gagaattgaa

1921 gaattcttta taactggtga gggccatttt aatgaagttt tacaatttag aacgccaggc

1981 acgttgagca ccacagagtc aacacctgct gggctgccaa cagctgaacc ttttaagtcc

2041 tacttcgcca aaggcttcct ctcgatagat tcaggttact actcagccaa atgttactca

2101 ggaacatcca attcagggct tcaattgatt aacattaccc gacattcaac tagaatagtt

2161 gacacacctg ggcctaagat cactaaccta aagaccatca actgcataaa cttgaaggca

2221 tcgatcttca aagaacatag agaggttgaa atcaatgtgc ttctccccca agttgcagtt

2281 aatctctcaa actgtcacgt tgtaatcaaa tcacatgtct gtgactactc tttagacatt

2341 gacggtgcgg tgaggcttcc tcacatttac catgaaggag ttttcatccc aggaacttac

2401 aaaatagtga tagataaaaa aaataagttg aatgacagat gcaccttatt taccgactgt

2461 gtgataaaag gaagggaggt tcgtaaagga cagtcagttt tgaggcagta caagacggaa

2521 atcaggattg gcaaggcatc aaccggcttt agaagattgc tttcagaaga acccagtgat

2581 gactgtgtat caagaactca actattaagg acagagactg cagagatcca cggcgacaac

2641 tatggtggcc cgggtgacaa aataaccatc tgcaatggct caactattgt agaccaaaga

2701 ctgggcagtg aactaggatg ctacaccatc aatagagtga ggtcattcaa gctatgcgaa

2761 aacagtgcca cagggaagaa ttgtgaaata gacagtgtcc cagttaaatg caggcagggt

2821 tattgcctaa gaatcactca ggaagggagg ggccacgtaa aattatctag gggctcagag

2881 gttgtcttag atgcatgcga tacaagctgt gaaataatga tacctaaggg cactggtgac

2941 atcctagttg actgttcagg tgggcagcaa cattttctaa aggacaattt gatagatcta

3001 ggatgcccca aaattccatt attgggcaaa atggctattt acatttgcag aatgtcaaac

3061 caccccaaaa caaccatggc tttcctcttc tggttcagct ttggctatgt aataacctgc

3121 atactttgca aggctatttt ttacttgtta ataattgttg gaacactagg gagaaggctc

3181 aagcagtata gagagttgaa acctcagact tgcaccatat gtgagacaac tcctgtaaat

3241 gcaatagatg ctgagatgca tgacctcaat tgcagttaca acatttgtcc ctactgtgca

3301 tctagactaa cctcagatgg gcttgctagg catgtgatac aatgccctaa gcggaaggag

3361 aaagtggaag aaactgaact gtacttgaac ttagaaagaa ttccttgggt tgtaagaaag

3421 ctgttgcagg tgtcagagtc aactggtgtg gcattgaaaa gaagcagttg gctgattgtg

3481 ctgcttgtgc tattcactgt ttcattatca ccagttcaat cagcacccat tggtcaaggg

3541 aagacaattg aggcataccg ggccagggaa gggtacacaa gtatatgcct ctttgtacta

3601 ggaagtatcc tatttatagt ttcttgccta atgaaagggc tggttgacag tgttggcaac

3661 tccttcttcc ctggactgtc catttgcaaa acgtgctcca taagcagcat taatggcttt

3721 gaaattgagt cccataagtg ctattgcagc ttattctgtt gcccctattg taggcactgc

3781 tctaccgata aagaaattca taagctgcac ttgagcatct gcaaaaaaag gaaaacagga

3841 agtaatgtca tgttggctgt ctgcaagctc atgtgtttca gggccaccat ggaagtaagt

3901 aacagagccc tgtttatccg tagcatcatc aacaccactt ttgttttgtg catactgata

3961 ctagcagttt gtgttgttag cacctcagca gtggagatgg aaaacctacc agcagggacc

4021 tgggaaagag aagaagacct aacaaatttc tgtcatcagg aatgccaggt tacagagact

4081 gaatgcctct gcccttatga agctctagta ctcagaaagc ctttattcct agatagtaca

4141 gctaaaggca tgaaaaatct gctaaattca acaagtttag aaacgagttt atcaattgag

4201 gcaccatggg gagcaataaa tgttcagtca acctacaaac caactgtgtc aactgcaaac

4261 atagcactca gttggagctc agtggaacac agaggcaata agatcttggt ttcaggcaga

4321 tcagaatcaa ttatgaagct ggaagaaagg acaggaatca gctgggatct cggtgtagaa

4381 gatgcctctg aatctaaact gcttacagta tctgtcatgg acttgtctca gatgtactct

4441 cctgtcttcg agtacttatc aggggacaga caggtggaag agtggcccaa agcaacttgc

4501 acaggtgact gcccagaaag atgtggctgc acatcatcaa cctgtttgca caaagaatgg

4561 cctcactcaa gaaattggag atgcaatccc acttggtgct ggggtgtagg gactggctgc

4621 acctgttgtg gattagatgt gaaagacctt tttacagatt atatgtttgt caagtggaaa

4681 gttgaataca tcaagacaga ggccatagtg tgtgtagaac ttactagtca ggaaaggcag

4741 tgtagcttga ttgaagcggg cacaaggttc aatttaggtc ctgtgaccat cacactgtca

4801 gaaccaagaa acatccaaca aaaactccct cctgaaataa tcacactgca tcctaggatc

4861 gaagaaggtt tCtttgacct gatgcatgtg caaaaggtgt tatcggcaag cacagtgtgt

4921 aagttgcaga gttgcacaca tggtgtgcca ggagacctac aggtctacca catcggaaat

4981 ttattaaaag gggataaggt aaatggacat ctaattcata aaattgagcc acacttcaac

5041 acctcctgga tgtcctggga tggttgtgac ctagactact actgcaacat gggagattgg

5101 ccttcttgca catacacagg ggtcacccaa cacaatcatg cttcatttgt aaacttactc

5161 aacattgaaa ctgattacac aaagaacttc cactttcact ctaaaagggt cactgcacac

5221 ggagatacac cacaactaga tcttaaggca agaccaacct atggtgcagg cgagatcact

5281 gttctggtag aagttgctga catggagtta catacaaaga agattgaaat atcaggctta

5341 aaatttgcaa gcttagcttg cacaggttgt tatgcttgta gctctagcat ctcatgcaaa

5401 gttagaattc atgtggatga accagatgaa cttacagtac atgttaaaag tgatgatcca

5461 gatgtggttg cagctagctc aagtctcatg gcaaggaagc ttgaatttgg aacagacagt

5521 acatttaaag ctttctcggc catgcctaaa acttctctat gtttctacat tgttgaaaga

5581 gaacactgta agagctgcag tgaagaagac acaaaaaaat gtgttaacac aaaacttgag

5641 caaccacaaa gcattttgat cgaacacaag ggaactataa tcggaaagca aaacagcact

5701 tgcacggcta aggcaagttg ctggttagag tcagtcaaga gtttCtttta tggcctaaag

5761 aacatgctta gtggcatttt tggcaatgtc tttatgggca ttttcttgtt ccttgccccc

5821 ttcatcctgt taatactatt ctttatgttt gggtggagga tcctattctg ctttaaatgt

5881 tgtagaagaa ccagaggcct gttcaagtat agacacctca aagacgatga agaaactggt

5941 tatagaagga ttattgaaaa actaaacaat aaaaaaggaa aaaacaaact gcttgatggt

6001 gaaagacttg ctgatggaag aattgccgaa ctgttctcta caaaaacaca cattggcACC

6061 CAGCTTTCTT GTACAAAGTG GTTCGATggg gatctagagg gcccgcggtt cgaaggtaag

6121 cctatcccta accctctcct cggtctcgat tctacgtaaG TCGACCTGCA G

**FEATURES Location/Qualifiers**

promoter 15..42

/note="p11 promoter"

CDS 43..762

/note="eGFP"

promoter 784..853

/note="mH5 promoter"

CDS 868..975

/note="tPA"

CDS 1009..6057

/dnas_title="envelope glycoprotein precursor"

/db_xref="GI:16271972"

misc_feature 6091..6114

/note="linker"

misc_feature 6115..6156

/note="V5"

CDS 868..6159

/note="tPA-GP-V5 fusion protein"

/translation="MDAMKRGLCCVLLLCGAVFVSPSQEIHARFRRGARSPIKQVCTK

KQAHISLMYAILCLQLCGLGETHGSHNETRHNKTDTMTTHGDNPSSEPPVSTALSITL

DPSTVTPTTPASGLEGSGEVYTSPPITTGSLPLSETTPELPVTTGTDTLSAGDVDPST

QTAGGTSAPTVRTSLPNSPSTPSTPQDTHHPVRNLLSVTSPGPDETSTPSGTGKESSA

TSSPHPVSNRPPTPPATAQGPTENDSHNATEHPESLTQSATPGLMTSPTQIVHPQSAT

PITVQDTHPSPTNRSKRNLKMEIILTLSQGLKKYYGKILRLLQLTLEEDTEGLLEWCK

RNLGLDCDDTFFQKRIEEFFITGEGHFNEVLQFRTPGTLSTTESTPAGLPTAEPFKSY

FAKGFLSIDSGYYSAKCYSGTSNSGLQLINITRHSTRIVDTPGPKITNLKTINCINLK

ASIFKEHREVEINVLLPQVAVNLSNCHVVIKSHVCDYSLDIDGAVRLPHIYHEGVFIP

GTYKIVIDKKNKLNDRCTLFTDCVIKGREVRKGQSVLRQYKTEIRIGKASTGFRRLLS

EEPSDDCVSRTQLLRTETAEIHGDNYGGPGDKITICNGSTIVDQRLGSELGCYTINRV

RSFKLCENSATGKNCEIDSVPVKCRQGYCLRITQEGRGHVKLSRGSEVVLDACDTSCE

IMIPKGTGDILVDCSGGQQHFLKDNLIDLGCPKIPLLGKMAIYICRMSNHPKTTMAFL

FWFSFGYVITCILCKAIFYLLIIVGTLGRRLKQYRELKPQTCTICETTPVNAIDAEMH

DLNCSYNICPYCASRLTSDGLARHVIQCPKRKEKVEETELYLNLERIPWVVRKLLQVS

ESTGVALKRSSWLIVLLVLFTVSLSPVQSAPIGQGKTIEAYRAREGYTSICLFVLGSI

LFIVSCLMKGLVDSVGNSFFPGLSICKTCSISSINGFEIESHKCYCSLFCCPYCRHCS

TDKEIHKLHLSICKKRKTGSNVMLAVCKLMCFRATMEVSNRALFIRSIINTTFVLCIL

ILAVCVVSTSAVEMENLPAGTWEREEDLTNFCHQECQVTETECLCPYEALVLRKPLFL

DSTAKGMKNLLNSTSLETSLSIEAPWGAINVQSTYKPTVSTANIALSWSSVEHRGNKI

LVSGRSESIMKLEERTGISWDLGVEDASESKLLTVSVMDLSQMYSPVFEYLSGDRQVE

EWPKATCTGDCPERCGCTSSTCLHKEWPHSRNWRCNPTWCWGVGTGCTCCGLDVKDLF

TDYMFVKWKVEYIKTEAIVCVELTSQERQCSLIEAGTRFNLGPVTITLSEPRNIQQKL

PPEIITLHPRIEEGFFDLMHVQKVLSASTVCKLQSCTHGVPGDLQVYHIGNLLKGDKV

NGHLIHKIEPHFNTSWMSWDGCDLDYYCNMGDWPSCTYTGVTQHNHASFVNLLNIETD

YTKNFHFHSKRVTAHGDTPQLDLKARPTYGAGEITVLVEVADMELHTKKIEISGLKFA

SLACTGCYACSSSISCKVRIHVDEPDELTVHVKSDDPDVVAASSSLMARKLEFGTDST

FKAFSAMPKTSLCFYIVEREHCKSCSEEDTKKCVNTKLEQPQSILIEHKGTIIGKQNS

TCTAKASCWLESVKSFFYGLKNMLSGIFGNVFMGIFLFLAPFILLILFFMFGWRILFC

FKCCRRTRGLFKYRHLKDDEETGYRRIIEKLNNKKGKNKLLDGERLADGRIAELFSTK

THIGTQLSCTKWFDGDLEGPRFEGKPIPNPLLGLDST"

//
